# Porous NASICON-Type Li_3_Fe_2_(PO_4_)_3_ Thin Film Deposited by RF Sputtering as Cathode Material for Li-Ion Microbatteries

**DOI:** 10.1186/s11671-016-1574-7

**Published:** 2016-08-17

**Authors:** Vinsensia Ade Sugiawati, Florence Vacandio, Marielle Eyraud, Philippe Knauth, Thierry Djenizian

**Affiliations:** 1 Aix-Marseille University, CNRS, MADIRELLaboratory, UMR 7246, 13397 Marseille, France; 2 Department of Flexible Electronics, Ecole National Supérieure des Mines de Saint-Etienne, Center of Microelectronics in Provence, 13 541 Gardanne, France

**Keywords:** Radio frequency sputtering, Li-ion microbatteries, NASICON-type Li_3_Fe_2_(PO_4_)_3_, Cathode thin film

## Abstract

We report the electrochemical performance of porous NASICON-type Li_3_Fe_2_(PO_4_)_3_ thin films to be used as a cathode for Li-ion microbatteries. Crystalline porous NASICON-type Li_3_Fe_2_(PO_4_)_3_ layers were obtained by radio frequency sputtering with an annealing treatment. The thin films were characterized by XRD, SEM, and electrochemical techniques. The chronoamperometry experiments showed that a discharge capacity of 88 mAhg^−1^ (23 μAhcm^−2^) is attained for the first cycle at C/10 to reach 65 mAhg^−1^ (17 μAhcm^−2^) after 10 cycles with a good stability over 40 cycles.

## Background

Due to remarkable properties such as excellent cycle life, high thermal resistance, no memory effect, and low self-discharge, Li-ion batteries have attracted great attention in electrochemical energy storage [1–3]. More recently, the miniaturization of these power sources has been investigated to meet many applications in microelectronics such as real-time clocking (RTC), radio frequency identification (RFID), sensors, medical implants, memory backup power, solar cell, and smartcards [4–6]. One of the major challenges in the microbattery field is related to the manufacturing process and its compatibility with the integrated circuit technology. Particularly, metallic Li which is currently used as a negative electrode is not compatible with the reflow soldering process because of its low melting point (180.5 °C). Therefore, the rocking chair technology involving stable materials must be explored. Many researches focused on the development of new components with nanostructured materials and 3D designs [7–11].

As potential cathode materials, the NASICON-type compounds A_3_Fe_2_(XO_4_)_3_ (A = Li, Na; X = P, As, S) with 3D frameworks like Li_3_Fe_2_(PO_4_)_3_ have attracted considerable attention [12]. NASICON-type Li_3_Fe_2_(PO_4_)_3_ can generate 2.8 V vs. Li with an excellent capacity retention, and up to 2 mol of Li^+^ can be reversibly intercalated into Li_3_Fe_2_(PO_4_)_3_, delivering a capacity of 128 mAhg^−1^. NASICON has also a relatively high ionic conductivity resulting from the disorder of lithium ions in the structure, favorable redox properties, low cost, structural stability, and simple fabrication procedures [5, 6, 12]. Many methods have been reported to synthesize Li_3_Fe_2_(PO_4_)_3_ nanostructures such as hydrothermal [13, 14], gel combustion [15], solid state [16–19], solution with the use of citric acid [20], sol-gel [21], ultrasonic spray combustion [22], and ion exchange with molten salt [23, 24].

In this work, we report the fabrication of porous NASICON-type Li_3_Fe_2_(PO_4_)_3_ thin film electrodes by radio frequency sputtering. Sputtering techniques are widely employed in microelectronics for the successive deposition of thin films; they are the main process used to fabricate planar all-solid-state Li-ion microbatteries [25, 26]. To the best of our knowledge, this approach has never been applied to deposit Li_3_Fe_2_(PO_4_)_3_. We show that this material tested as a cathode reveals a good electrochemical behavior for all-solid-state Li-ion microbatteries.

## Methods

### Preparation of Li_3_Fe_2_(PO_4_)_3_ Thin Films

Firstly, thin films of LiFePO_4_ were deposited on titanium foil as substrate by radio frequency (RF) sputtering (PLASSYS). The target was LiFePO_4_ (purity 99.9 %, Neyco). The process was carried out in a vacuum chamber with a base pressure before deposition of 10^−6^ Torr. The sputtering gas was pure argon, and the working pressure was 10 mTorr with a gas flow of 21.5 sccm. A sputtering power of 150 W was applied to the target. The deposition time was 3 h. Secondly, the as-deposited thin films were annealed in air at 700 °C for 3 h with a heating rate of 10 °C min^−1^ (furnace: NABERTHERM Controller B 180).

### Structural Characterization

Phases and crystallinity of thin films were examined by X-ray diffraction (XRD) with Cu Kα radiation (wavelength = 1.5405 Å) using a D5000 BRUKER-SIEMENS diffractometer. The morphology of the Li_3_Fe_2_(PO_4_)_3_ was investigated by scanning electron microscopy (SEM, Hitachi, S-570). Thickness measurement was performed by profilometry analysis using a DEKTAT XT (BRUKER) equipment. A portion of the sample is masked during deposition, and the thickness is given by the height of the step.

### Electrochemical Measurements

The electrochemical tests of the half-cell were performed using Swagelok cell assembled in a glove box filled with purified argon in which moisture and oxygen content were less than 0.5 ppm. The half-cells consisted of metallic Li as counter electrode. Two circular sheets of Whatman glass microfiber soaked with lithium hexafluorophosphate in ethylene carbonate and diethylene carbonate (1 M LiPF_6_ in (EC:DEC) 1:1 *w*/*w*) were used as separator. Cyclic voltammetry measurements were performed using a Versastat potentiostat (AMETEK) in the range of 1.25–5 V vs. Li/Li^+^ with a scan rate of 1 mVs^−1^. Galvanostatic cycles were performed with a VMP3 (Bio Logic) at 25 °C between 2 and 4 V vs. Li/Li^+^. The different cycles of the charge/discharge rate were investigated at a kinetic rate of C/10 and C/5 considering that the theoretical capacity of Li_3_Fe_2_(PO_4_)_3_ is 128.25 mAhg^−1^. Hence, the applied currents were 3.3 and 6.6 μA cm^−2^, respectively. No additives or binder was used for all electrochemical tests.

## Results and Discussion

**Fig. 1:**
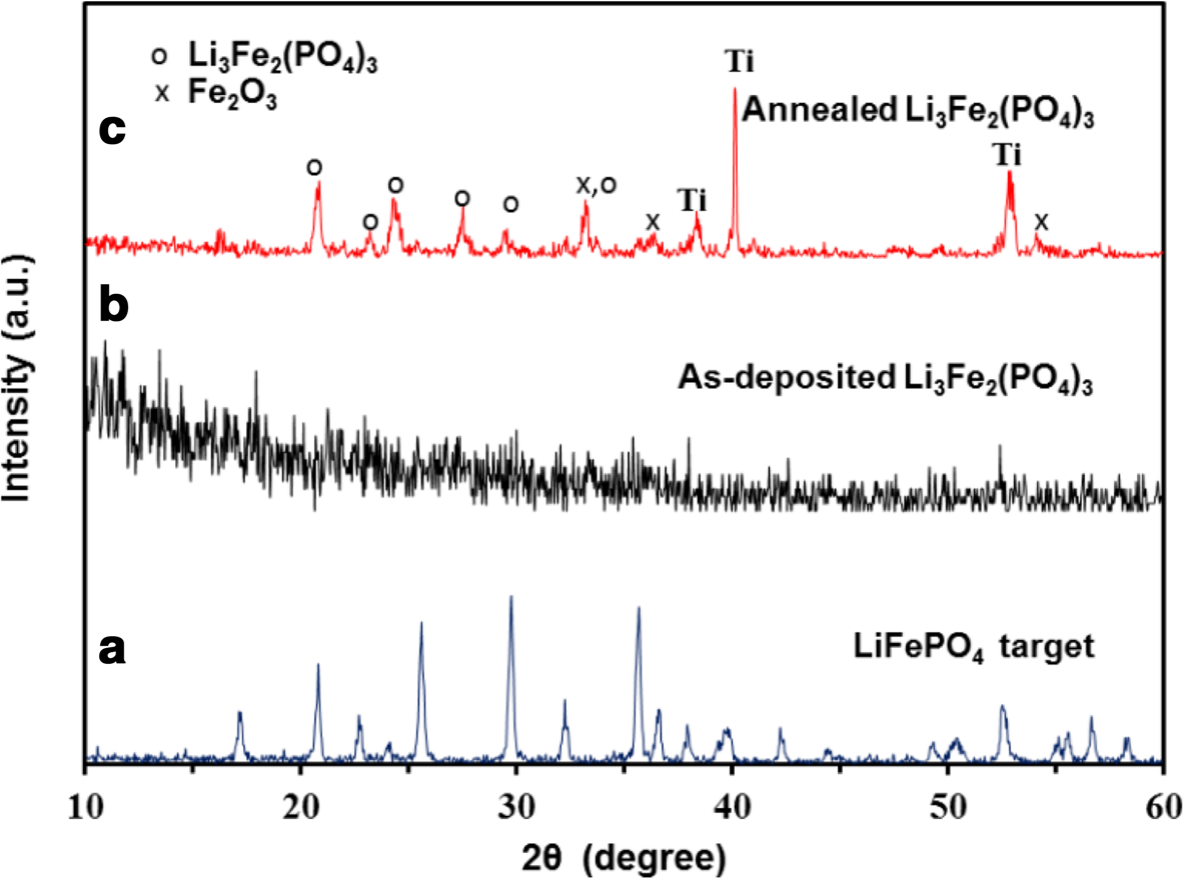
XRD patterns of samples **a** LiFePO_4_ target, **b** as-deposited Li_3_Fe_2_(PO_4_)_3_, and **c** annealed Li_3_Fe_2_(PO_4_)_3_ at 700 °C for 3 h

Prior to battery assembly, the crystallinity of the thin films were examined by XRD. The XRD patterns of initial target and as-deposited sample are shown in Fig. 1. It can be seen that the target is well-crystallized and corresponds to the Olivine-type LiFePO_4_ phase (JCPDS file no. 040-1499). Various deposition parameters (such as target power, substrate temperature, or argon pressure) were previously investigated, and in all cases, the as-deposited thin films were amorphous. As it can be seen in Fig. 1, the annealing treatment leads to the crystallization of NASICON-type Li_3_Fe_2_(PO_4_)_2_ phases (JCPDS file no. 047-0107), which is known to have better electrochemical properties [27]. Three small peaks are attributable to the Fe_2_O_3_ phase. The oxidation state of Fe in LiFePO_4_ and Li_3_Fe_2_(PO_4_) are +II and +III, respectively. It can be noted that the annealing in air of amorphous LiFePO_4_ not only leads to crystallization but also to the iron oxidation according to (Eq. 1) [28, 29]:

1 $$ 12{\mathrm{Li}\mathrm{FePO}}_4+3{\mathrm{O}}_2=4{\mathrm{Li}}_3{\mathrm{Fe}}_2{\left({\mathrm{PO}}_4\right)}_3 + 2{\mathrm{Fe}}_2{\mathrm{O}}_3 $$

However, the conversion reaction of Fe_2_O_3_ with Li^+^ occurs at low potential which is not interesting for its use as a cathode [30, 31].

**Fig. 2:**
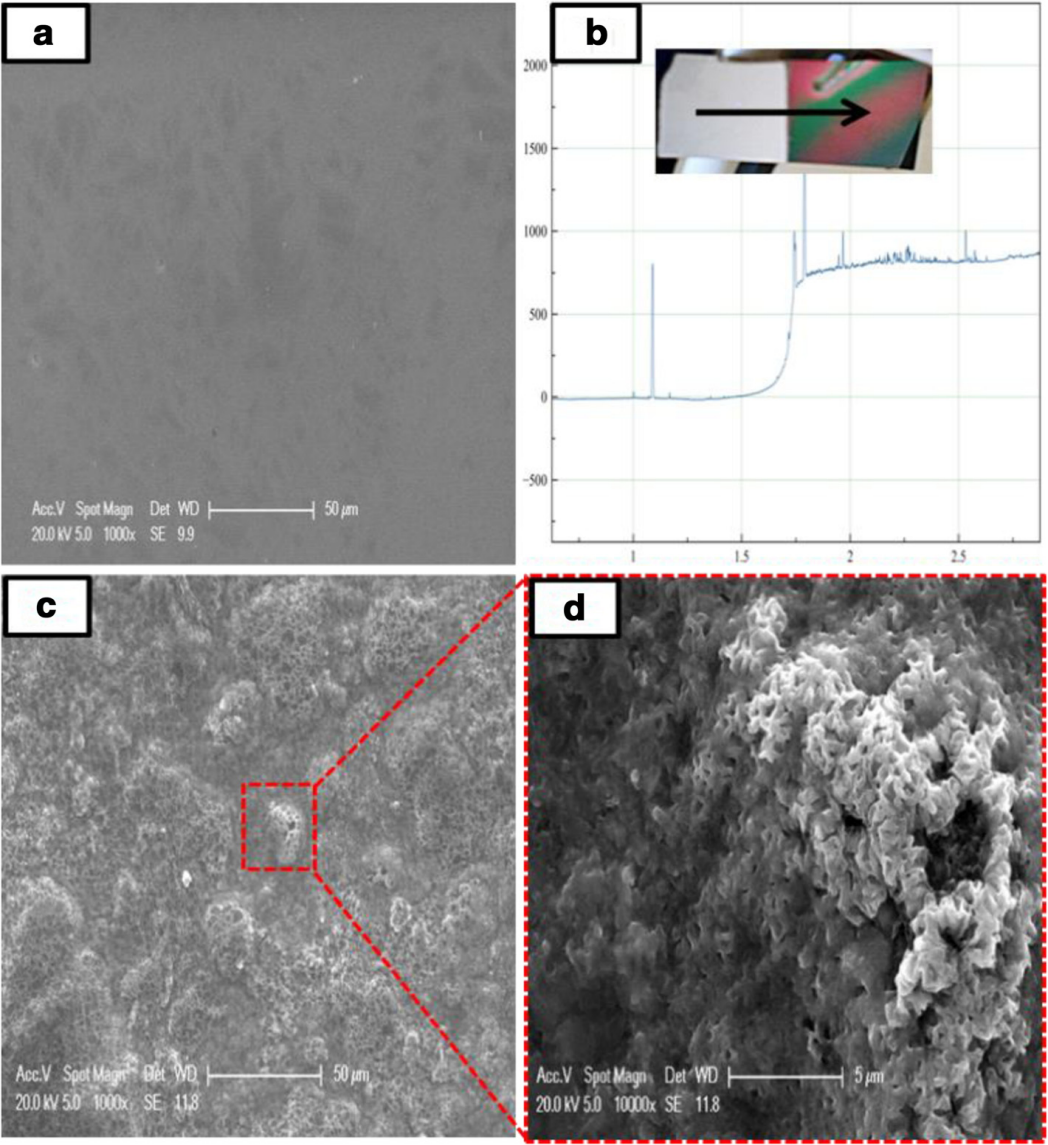
**a** as-deposited Li_3_Fe_2_(PO_4_)_3_ with **b** profilometry analysis, low (**c**) and high magnification (**d**) SEM images of porous Li_3_Fe_2_(PO_4_)_3_ deposited by RF sputtering after annealing treatment

The morphology of the Li_3_Fe_2_(PO_4_)_3_ thin films before and after annealing was observed by SEM. From Fig. 2a, it is apparent that the as-deposited Li_3_Fe_2_(PO_4_)_3_ layer is homogeneous with a thickness of 850 nm according to the surface profilometry analysis given in Fig. 2b. After annealing, the film became rough and a sponge-like texture clearly appeared (Fig. 2c, d). Hence, the thermal treatment promoted the formation of a mesoporous material with a larger surface area than the as-deposited layer.

**Fig. 3:**
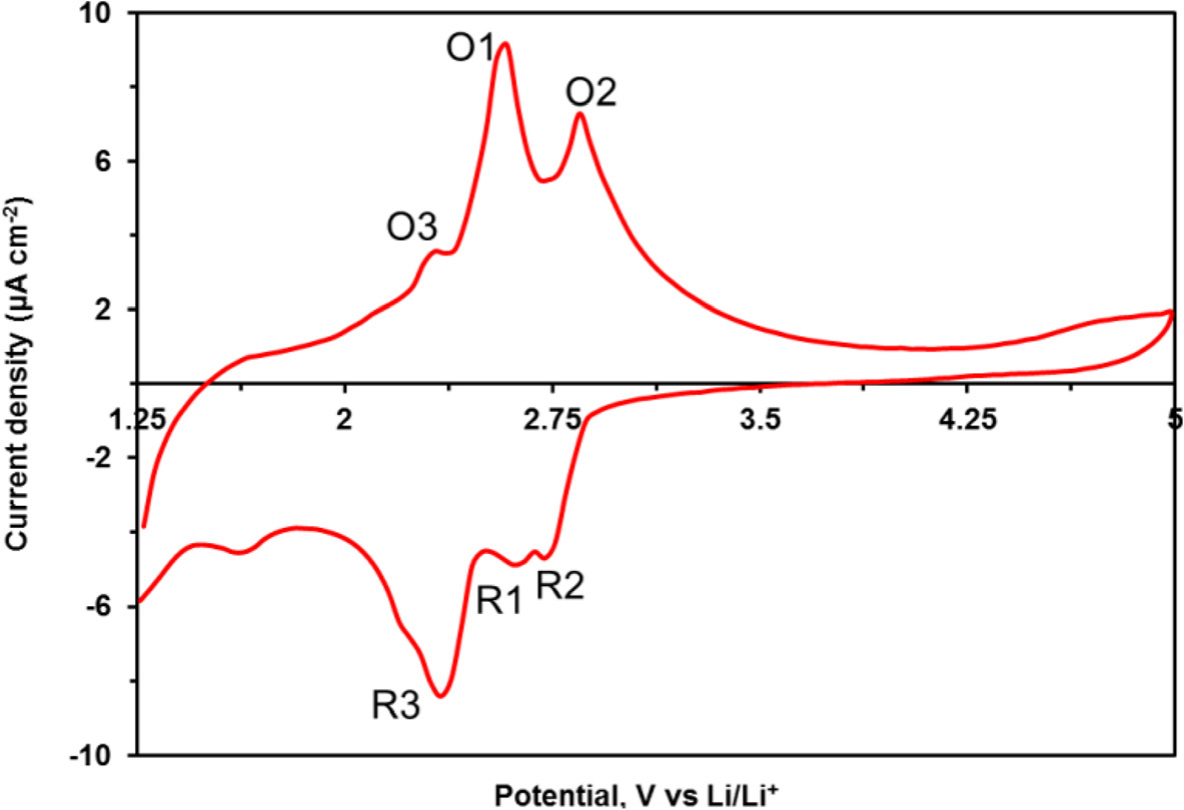
CV curve of the annealed Li_3_Fe_2_(PO_4_)_3_ thin film deposited on Ti substrate recorded at a scan rate of 1 mVs^−1^

The electrochemical characterization of the porous Li_3_Fe_2_(PO_4_)_3_ thin film was carried out by cyclic voltammetry (CV). Figure 3 shows the cyclic voltammogram (CV) is obtained. In agreement with literature, the electrochemical reactivity of crystalline Li_3_Fe_2_(PO_4_)_3_ is confirmed by the presence of anodic and cathodic peaks [3, 32]. These two peak pairs can be attributed to the reversible insertion reactions of two Li^+^ according to Eqs. (2) and (3):

- First reduction (R1 2.68 V/Li) and oxidation peaks (O1 2.58 V/Li)

2 $$ {\mathrm{Li}}_4{\mathrm{Fe}}_2{\left({\mathrm{PO}}_4\right)}_3={\mathrm{Li}}^{+}+{\mathrm{Li}}_3{\mathrm{Fe}}_2{\left({\mathrm{PO}}_4\right)}_3+{\mathrm{e}}^{-} $$

- Second reduction (R2 2.72 V/Li) and oxidation peaks (O2 2.85 V/Li)

3 $$ {\mathrm{Li}}_5{\mathrm{Fe}}_2{\left({\mathrm{PO}}_4\right)}_3={\mathrm{Li}}^{+}+{\mathrm{Li}}_4{\mathrm{Fe}}_2{\left({\mathrm{PO}}_4\right)}_3+{\mathrm{e}}^{-} $$

The presence of an additional reversible peak (O3 and R3 peaks) at around 2.4 V reveals that Li^+^ can also react with another phase. This active material is supposed to be LiFePO_4_(OH) into which lithium intercalation occurs through the reduction of Fe^3+^ to Fe^2+^ [33–35]. Compared with literature, it can be noticed that the peaks are quite broad. This phenomenon can be explained by the presence of pores. Indeed, the large surface area offered by the porous texture promotes the storage of charge at the surface according to a non-faradaic process. Hence, the contribution of the capacitive effect leads to the broadening of the peaks.

**Fig. 4:**
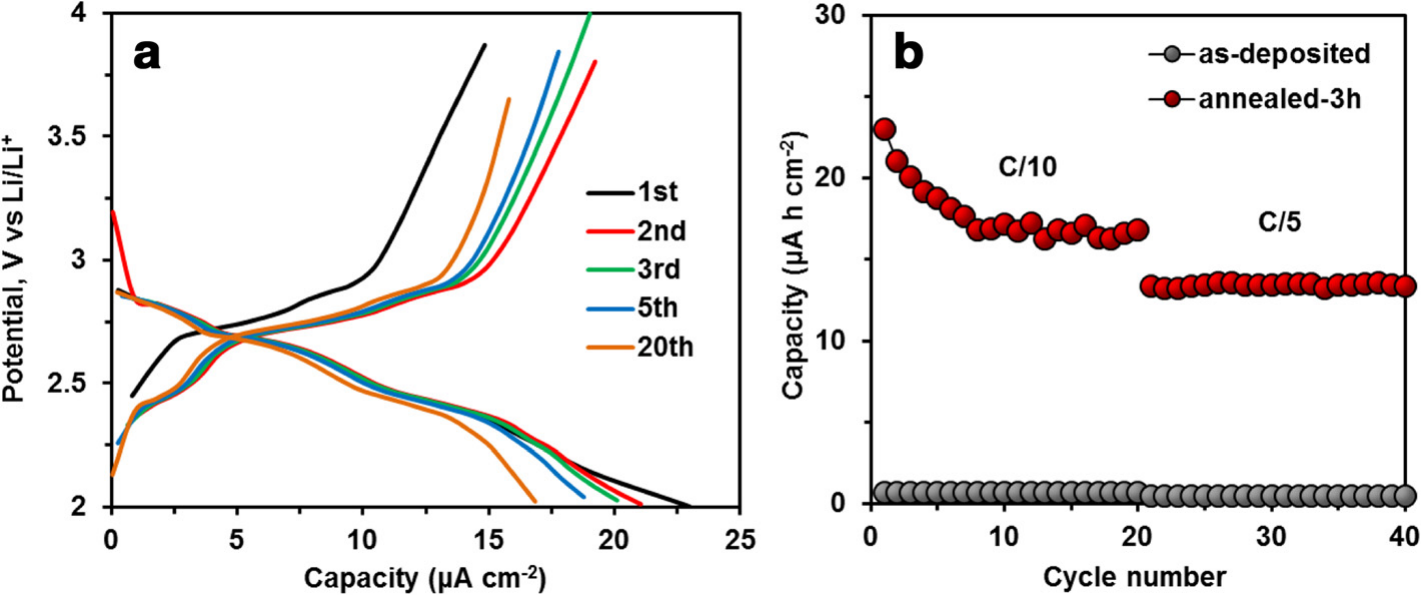
Battery cycling tests at the kinetic rate of C/10 in the potential window of 2–4 V (**a**). Discharge capacity of cathode at multi-C-rate (**b**)

The galvanostatic charge/discharge profiles obtained at C/10 of the half-cell battery are shown in Fig. 4a. In agreement with the CV curve, the presence of three pseudo-plateaus confirms that Li^+^ ions react with Li_3_Fe_2_(PO_4_)_3_ and LiFePO_4_(OH).

The capacity values obtained for the as-deposited sample (Fig. 4b) are very low in the range of 1–2 μAhcm^−2^. But, a first discharge capacity of 88 mAhg^−1^ (23 μAhcm^−2^) is achieved at C/10 for the annealed sample. An average value of about 65 mAhg^−1^ (17 μAhcm^−2^) is delivered after 20 cycles. This capacity corresponds to more than 50 % of the theoretical capacity of Li_3_Fe_2_(PO_4_)_3_ (33 μAhcm^−2^). We can observe that capacity is slightly fading during the first cycles. The capacity lost may be caused by the different effects which are associated with side reactions due to the phase changes in the insertion electrode, dissolution of the active material, or the decomposition of the electrolyte. The capacity loss has been also discussed by Song et al. [36] who reported that capacity fading can be attributed to surface impurities (such as lithiated iron oxide which is also involved in the lithium insertion/extraction) leading to the irreversible reaction of Li^+^ with iron oxide and the formation of a solid electrolyte interface (SEI) at the surface of the electrode.

In order to study the operational stability of the NASICON-type Li_3_Fe_2_(PO_4_)_3_, the cycling life performance has been also studied at two different C-rates (Fig. 4b). From this graph, it can be seen that the capacity becomes stable after 10 cycles. At C/5, the battery capacity is about 50 mAhg^−1^ (13 μAhcm^−2^) and remains constant over 40 cycles. These results also show that the porous geometry of the mesoporous electrode is beneficial for tolerating the volume expansion that generally occur during alloying/de-alloying reactions.

## Conclusions

In this work, we report the fabrication of mesoporous NASICON-type Li_3_Fe_2_(PO_4_)_3_ thin film by radio frequency sputtering. The electrochemical studies suggest that after annealing, the crystallized layer is also composed of LiFePO_4_(OH), which is able to react reversibly with Li^+^. In the first cycle, the discharge capacity reaches about 88 mAhg^−1^ (23 μAhcm^−2^) at C/10 and attains 65 mAhg^−1^ (17 μAhcm^−2^) after 10 cycles. The electrochemical characterizations also reveal a good stability suggesting that this material can be used as a cathode for Li-ion microbatteries.

## References

[1] Padhi AK, Nanjundaswamy KS, Goodenough JB (1997). Phospho-olivines as positive-electrode materials for rechargeable lithium batteries. J Electrochem Soc.

[2] Li D, Zhou H (2014). Two-phase transition of Li-intercalation compounds in Li-ion batteries. Mater Today.

[3] Xu J, Deshpande RD, Pan J (2015). Electrode side reactions, capacity loss and mechanical degradation in lithium-ion batteries. J Electrochem Soc.

[4] Porthault H, Decaux C (2016). Electrodeposition of lithium metal thin films and its application in all-solid-state microbatteries. Electrochim Acta.

[5] Luais E, Ghamouss F, Wolfman J (2015). Mesoporous silicon negative electrode for thin film lithium-ion microbatteries. J Power Sources.

[6] Lafont U, Anastasopol A, Garcia-Tamayo E, Kelder E (2012). Electrostatic spray pyrolysis of LiNi_0.5_Mn_1.5_O_4_ films for 3D Li-ion microbatteries. Thin Solid Films.

[7] Ferrari S, Loveridge M, Beattie SD (2015). Latest advances in the manufacturing of 3D rechargeable lithium microbatteries. J Power Sources.

[8] Meng X, Yang X-Q, Sun X (2012). Emerging applications of atomic layer deposition for lithium-ion battery studies. Adv Mater.

[9] Plylahan N, Kyeremateng N, Eyraud M (2012). Highly conformal electrodeposition of copolymer electrolytes into titania nanotubes for 3D Li-ion batteries. Nanoscale Res Lett.

[10] Plylahan N, Maria S, Phan TN (2014). Enhanced electrochemical performance of lithium-ion batteries by conformal coating of polymer electrolyte. Nanoscale Res Lett.

[11] Ellis BL, Knauth P, Djenizian T (2014). Three-dimensional self-supported metal oxides for advanced energy storage. Adv Mater.

[12] Masquelier C, Padhi AK, Nanjundaswamy KS, Goodenough JB (1998). New cathode materials for rechargeable lithium batteries: the 3-D framework structures Li_3_Fe_2_(XO_4_)_3_(X = P, As). J Solid State Chem.

[13] Sato M (2002). Preparation of iron phosphate cathode material of Li_3_Fe_2_(PO_4_)_3_ by hydrothermal reaction and thermal decomposition processes. Solid State Ionics.

[14] Yang S, Zavalij PY, Stanley Whittingham M (2001). Hydrothermal synthesis of lithium iron phosphate cathodes. Electrochem Commun.

[15] Karami H, Taala F (2011). Synthesis, characterization and application of Li_3_Fe_2_(PO_4_)_3_ nanoparticles as cathode of lithium-ion rechargeable batteries. J Power Sources.

[16] Morcrette M, Wurm C, Masquelier C (2002). On the way to the optimization of Li_3_Fe_2_(PO_4_)_3_ positive electrode materials. Solid State Sci.

[17] Pylinina AI, Mikhalenko II, Ivanov-Shits AK (2006). The influence of plasma chemical treatments on the activity of the Li_3_Fe_2_(PO_4_)_3_ catalyst in butanol-2 transformations. Russ J Phys Chem.

[18] Smiley DL, Davis LJM, Goward GR (2013). An improved understanding of Li^+^ hopping pathways and rates in Li_3_Fe_2_(PO_4_)_3_ using selective inversion ^6^Li NMR spectroscopy. J Phys Chem C.

[19] Kim HS, Kim CS (2013). Spin ordering between sub-lattices in nasicon Li_3_Fe_2_(PO_4_)_3_ measured by Mössbauer spectroscopy. J Appl Phys.

[20] Shirakawa J, Nakayama M, Wakihara M, Uchimoto Y (2006). Changes in electronic structure upon Li insertion reaction of monoclinic Li_3_Fe_2_(PO_4_)_3_. J Phys Chem B.

[21] Salah AA, Jozwiak P, Garbarczyk J (2005). Local structure and redox energies of lithium phosphates with olivine- and nasicon-like structures. J Power Sources.

[22] Ivanov-Schitz A (2001). Li_3_Fe_2_(PO_4_)_3_ solid electrolyte prepared by ultrasonic spray pyrolysis. Solid State Ionics.

[23] Andersson A (2001). Lithium insertion into rhombohedral Li_3_Fe_2_(PO_4_)_3_. Solid State Ionics.

[24] Becht G, Hwu S-J (2006). Hydrothermal ion exchange on submillimeter-size single crystals of a new iron(III) phosphate. Chem Mater.

[25] Yoon Y, Park C, Kim J, Shin D (2012). Characterization of lithium borophosphate glass thin film electrolytes deposited by RF-magnetron sputtering for micro-batteries. Solid State Ionics.

[26] Nakazawa H, Sano K, Baba M, Kumagai N (2014). Stability of thin-film lithium-ion rechargeable batteries fabricated by sputtering method without heating. J Electrochem Soc.

[27] Bajars G, Kucinskis G, Smits J, Kleperis J (2011). Physical and electrochemical properties of LiFePO_4_/C thin films deposited by direct current and radiofrequency magnetron sputtering. Solid State Ionics.

[28] Hamelet S, Gibot P, Casas-Cabanas M (2009). The effects of moderate thermal treatments under air on LiFePO_4_-based nano powders. J Mater Chem.

[29] Yu F, Zhang L, Li Y (2014). Mechanism studies of LiFePO _4_ cathode material: lithiation/delithiation process, electrochemical modification and synthetic reaction. RSC Adv.

[30] Larcher D, Masquelier C, Bonnin D (2003). Effect of particle size on lithium intercalation into α-Fe_2_O_3_. J Electrochem Soc.

[31] Ortiz GF, Hanzu I, Lavela P (2010). A novel architectured negative electrode based on titania nanotube and iron oxide nanowire composites for Li-ion microbatteries. J Mater Chem.

[32] Antolini E (2004). LiCoO_2_: formation, structure, lithium and oxygen nonstoichiometry, electrochemical behaviour and transport properties. Solid State Ionics.

[33] Yaghoobnejad Asl H, Choudhury A (2014). Phosphorous acid route synthesis of iron tavorite phases, LiFePO_4_(OH)_x_ F_1−x_ [0 ≤ × ≤ 1] and comparative study of their electrochemical activities. RSC Adv.

[34] Ellis BL, Ramesh TN, Rowan-Weetaluktuk WN (2012). Solvothermal synthesis of electroactive lithium iron tavorites and structure of Li_2_FePO_4_F. J Mater Chem.

[35] Marx N, Croguennec L, Carlier D (2010). The structure of tavorite LiFePO_4_(OH) from diffraction and GGA + U studies and its preliminary electrochemical characterization. Dalton Trans.

[36] Song J, Cai M-Z, Dong Q-F (2009). Structural and electrochemical characterization of SnO_x_ thin films for Li-ion microbattery. Electrochim Acta.

